# Vitamin D Deficiency and Insufficiency in Obese Children and Adolescents and Its Relationship with Insulin Resistance

**DOI:** 10.1155/2013/631845

**Published:** 2013-03-27

**Authors:** Emel Torun, Erdem Gönüllü, İlker Tolga Özgen, Ergül Cindemir, Faruk Öktem

**Affiliations:** ^1^Department of Pediatrics, Medical Faculty, Bezmialem Vakif University, Fatih 3409, Istanbul, Turkey; ^2^Department of Pediatric Endocrinology and Metabolism, Medical Faculty, Bezmialem Vakif University, Fatih 3409, Istanbul, Turkey; ^3^Department of Pediatric Nephrology, Medical Faculty, Bezmialem Vakif University, Fatih 3409, Istanbul, Turkey

## Abstract

*Objectives*. We aimed to determine the relationship between insulin resistance and serum 25-hydroxyvitamin D (25-OHD) levels in obese children and their nonobese peers. *Materials and Methods*. Included in the study group were 188 obese children (aged 9–15 years), and 68 age- and gender-matched healthy children of normal weight as control group. Anthropomorphic data were collected on patients and fasting serum glucose, insulin, serum lipids, alanine aminotransaminase (ALT) and 25-OHD were measured. The homeostatic model assessment of insulin resistance (HOMA-IR) was calculated in both groups. *Results*. The levels of 25-OHD in the obese group were significantly lower than those of the nonobese (*P* = 0.002). HOMA-IR, triglycerides, low-density lipoprotein, and ALT levels in the obese group were significantly higher than values of control group (*P* < 0.001 and *P* = 0.002, resp.). In the obese group, vitamin D deficiency, insufficiency, and sufficiency (25-OHD < 10 ng/dl, < 20, >10 ng/dl; > 20 ng/dl, resp.) were not correlated with HOMA-IR (*r* : −0.008, *P* = 0.935). HOMA-IR was negatively correlated with BMI, BMI SDS, and BMI%, and triglycerides, low-density lipoprotein, and ALT levels (*P* < 0.001). *Conclusion*. The insulin resistance of the obese subjects who were vitamin D deficient and insufficient did not statistically differ from those with vitamin D sufficiency. Low 25-hydroxyvitamin D levels were not related with higher insulin resistance in obese children and adolescents. In obese subjects, insulin resistance was affected more from BMI, BMI SDS, and BMI% than from 25-hydroxyvitamin D levels.

## 1. Introduction

Vitamin D deficiency, a pandemic health problem, is a major cause of rickets in infants and toddlers and of osteopenia in adolescents [1–6]. The production of vitamin D in the skin depends on sunshine exposure, latitude, skin-covering clothes, the use of sun block, and skin pigmentation. Although the Mediterranean region generally has a sunny climate, higher rates of hypovitaminosis D are seen in European and Mediterranean countries [7–10]. Vitamin D deficiency is especially common in the Middle East because of the prevalence of wearing skin-covering clothes and because of staying out of the sun [11].

Besides acting as a regulatory hormone in calcium metabolism, noncalciotropic effects of vitamin D such as cellular differentiation and replication in many organs have been found. Vitamin D is also critical in glucose homeostasis and insulin secretion via its endocrine mechanisms [12–14], besides its autocrine and paracrine role in adipocytes [15, 16]. Insulin resistance plays a major role in obesity, and as a population gets heavier at younger ages, the age of onset of non-insulin-dependent diabetes mellitus also decreases [17]. Regrettably, obesity and adiposity is an emerging trend in the industrialized world it is a result of alimited exercise, a sedentary lifestyle, and replete diets with high-calorie, low-nutrient foods.

 The literature provides conflicting data as to whether vitamin D deficiency and insufficiency is a risk factor for the development of impaired glucose tolerance in childhood obesity [18]. Low 25-hydroxyvitamin D level is found to be associated with insulin resistance in adults [19], but no relationship was found with parameters of glucose homeostasis and insulin sensitivity in healthy youth [20]. This study was conducted to study the relationship of vitamin D deficiency and insufficiency with insulin resistance in obese children and adolescents and the prevalence of vitamin D deficiency among children living in the metropolitan area of Istanbul, Turkey.

## 2. Materials and Methods

Enrolled in the study were 118 obese children (50% males, 50% females; mean age 12 ± 2.2 years) and a control group of 68 healthy nonobese children (48.5% males, 51.5% females; mean age 12.6 ± 1.7 years). They had been admitted to the Bezmialem Vakif University General Pediatrics Clinic in the Marmara region between January 2011 and January 2012.

Each participant underwent a detailed physical examination (including evaluation for syndromes and endocrine diseases), as well as a laboratory evaluation. Standing height was measured to the nearest 0.1 cm with a Harpenden fixed stadiometer. Body weight (kg) was measured on a SECA balance scale to the nearest 0.1 kg, with each subject dressed in a light T-shirt and shorts. Body mass index (BMI) was calculated by dividing weight by height (kg/m²). Obesity was defined as the BMI > 97th percentile, the definition of the International Task Force of Obesity in Childhood and Population-Specific Data [21, 22]. Waist circumference was measured between the xiphoid process and iliac crest. Children whose obesity was the result of a syndromal problem (Prader Willi, Laurence-Moon Biedl syndrome, etc.) were excluded, as were those whose obesity had an endocrinal cause such as Cushing's Syndrome or hypothyroidism. None of the participants was using meneedication or had a history or evidence of current metabolic, cardiovascular, respiratory, or hepatic disease. Patients taking vitamin and/or mineral supplements were excluded.

All blood analyses were performed on fasting samples in both the study and control groups. Cholesterol, high-density lipoprotein (HDL), low-density lipoprotein (LDL), and triglycerides were measured by the homogeneus colorimetric enzyme technique (Roche, Cobas 8000). Serum 25-OHD levels were determined by the electrochemiluminescence enzyme immunoassay method (ECLIA) (ADVIA Centaur, USADPC Co., USA). Glucose was measured by the glucose oxidase technique (Siemens ADVIA 1800) and insulin levels were analyzed with direct chemiluminescence technique (Siemens centaur, USA). Insulin resistance was estimated from fasting plasma measurements using HOMA-IR (insulin (mU/L) × glucose (mmol/L)/22.5) [23]. Insulin resistance criteria were HOMA-IR >2.5 for prepubertal children and HOMA-IR >4.0 for adolescents [24]. Vitamin D status was classified as either deficient, insufficient, or sufficient (serum 25-OHD: <10 ng/mL, 10–20 ng/mL, and >20 ng/mL, resp.) [25].

Statistical analysis was performed with PASW Statistics, v.13.0. A paired *t*-test was used to calculate the difference of two parameters in groups; one-way ANOVA was used in the calculation of difference of two parameters in groups with more than two in the same group and between different groups. Multiple comparisons were done with the Pearson correlation. Categorical data were evaluated using the Chi-square test; *P* < 0.05 was accepted as statistically significant.

The study was approved by the local ethical committee. Written informed consent was obtained from parents.

## 3. Results

Age and gender distribution were not statistically different between the two groups (*P* = 0.79 and 0.839, resp.). Subjects of the obese group had significantly higher waist circumference SDS (0.027), BMI (*P* < 0.001), BMI SDS (*P* > 0.001), and BMI percentage (*P* > 0.001) compared with the control group (Table 1).

Table 2 presents the biochemical characteristics of all subjects. Compared with the nonobese subjects, obese subjects had higher fasting insulin, HOMA-IR, serum ALT, triglyceride, and total and LDL-cholesterol levels. The 25-OHD levels of obese children were significantly lower than those of the non-obese (*P* = 0.002). HDL-cholesterol levels of the obese subjects were significantly lower than the non-obese ones.

The obese group was classified according to 25-OHD levels as vitamin D deficient **(**≤10 ng/mL), insufficient (10–20 ng/mL), and sufficient (25-OHD > 20 ng/dL) [26]. The groups were similar in age and gender ratio. The correlation of vitamin D levels with insulin resistance in the obese group was evaluated with HOMA-IR. The HOMA-IR levels of the obese subjects who were vitamin D deficient and insufficient did not statistically differ from those with vitamin-D-sufficient ones (*P* = 0.72) (Table 3).

Table 4 presents the correlations among HOMA-IR with other biochemical parameters in the obese group. HOMA-IR was not correlated significantly with age and serum 25-OHD levels but significantly correlated with triglyceride and LDL levels. HOMA-IR correlated significantly with BMI (*P* < 0.001), BMI SDS (*P* < 0.01), and BMI percentage (*P* < 0.001).

## 4. Discussion

Vitamin D deficiency and insufficiency are epidemic but commonly undiagnosed among obese children. Obese children in turn have higher risk of hypovitaminosis D. Turkey's vitamin D prophylaxis augmentation program (started in 2005) has resulted in a marked decrease in vitamin D deficiency in healthy children under 1 year of age [26]; yet despite these improvements, most Turkish adolescents are still vitamin D deficient. In our study, 25-OHD levels were significantly lower in the obese group compared with healthy subjects. This finding revealed that obesity could be a risk factor of hypovitaminosis D in Turkish children and adolescents. Recent studies from different countries have also demonstrated that vitamin D deficiency is common in obese children [27], possibly due to the low quality of diet [28]. Here in Turkey, rates of hypovitaminosis D in healthy adolescents were 59.4% and 65% in two different studies [26, 29].

 Vitamin D plays an important role in glucose homeostasis in the mechanism of insulin release. Most of the studies suggested vitamin D deficiency as a risk factor of disturbed glucose homeostasis in adults [30–33], but this hypothesis is still controversial in relation to children. It remains unclear if vitamin D deficiency and insufficiency are associated with insulin resistance in obese children and adolescents.

 Insulin resistance was estimated from fasting plasma measurements using HOMA-IR. In our study, insulin resistance values were higher in obese subjects compared with the healthy, vitamin-D-sufficient, age- and gender-matched children and adolescents. In order to determine the role of 25-hydroxyvitamin D levels in glucose intolerance and insulin resistance in obese children, we compare the HOMA-IR levels of vitamin-D-sufficient obese children and adolescents with the vitamin-D-deficient and -insufficient obese ones. There were no significant differences in HOMA-IR levels in obese children and adolescents due to 25-hydroxyvitamin D levels. In correlation analyses, we found that HOMA-IR depended on the degree of obesity and correlated with serum lipid profile. There was a significant trend towards higher insulin concentrations and insulin resistance in subjects with higher body mass index independent from 25-hydroxyvitamin D levels.

 Recent studies researching the relationship between vitamin D deficiency and insulin resistance in obese children revealed controversial results. In two studies, no relationship was found between low vitamin D status and insulin resistance [34, 35]. On the other hand, the literature mostly supported the hypothesis that low vitamin D status is associated with worse glucose tolerance. Kelly et al. [36] showed that vitamin D deficiency is associated with increased insulin resistance in children. Alemzadeh et al. [37] observed a positive relationship between vitamin D status and insulin sensitivity in children. The study by Reis et al. [16] showed that low serum vitamin D in adolescents was strongly associated with increased risk for fasting hyperglycemia, hypertension, and metobolic syndrome. In the study conducted by Garanty-Bogacka et al., fasting insulin levels and HOMA-IR were found correlated with low vitamin D levels [38].

 A limitation of this study is that vitamin D deficiency and insufficiency was defined only by 25-OHD levels. It is 1,25-dihydroxyvitamin D which is active on the vitamin D receptor in the insulin producing cells. It was also, not possible to evaluate other parameters modifying bone health such as subjects' dietary calcium intake or the responsiveness of the vitamin D receptor. In addition, with the lack of consensus regarding the definition of optimal vitamin D status in children, it is not possible to determine the level of vitamin D which disturbs glucose homeostasis and causes metabolic effects.

 In conclusion, in our study, insulin resistance correlated mostly with BMI but not with 25-hydroxyvitamin D levels in the obese children and adolescents. We found significant association between the degree of obesity and some biochemical parameters with insulin resistance, but different levels of 25 hydroxyvitamin D status among obese children were not an independent predictor of insulin resistance.

## Figures and Tables

**Table 1:** Mean anthropometric data of the study and control groups.

	Obese (*n* = 118)	Nonobese (*n* = 68)	*P*
Male/female	59/59	33/35	0.839
Age (years ± SD)	12 ± 2.2	12.6 ± 1.7	0.79
Waist circumference (cm)	87.1 ± 10.6	68.2 ± 14.7	0.027
BMI (kg/m²)	29.6 ± 3.9	20.1 ± 3	<0.001
BMI SDS	2.1 ± 0.2	0.15 ± 1	<0.001
BMI%	97.8 ± 1.1	54 ± 29.3	<0.001

SD: standard deviation, BMI: body mass index, BMI SDS: body mass index standard deviation score, BMI%: body mass index percentage.

**Table 2:** Metabolic markers of the study and control groups.

	Obese group (*n* = 118)	Nonobese group (*n* = 68)	*P*
Glucose (mg/dL)	91.2 ± 10.5	89.1 ± 10.4	0.18
Insulin (*μ*U/mL)	17.4 ± 10.2	14.1 ± 8.3	0.024
HOMA-IR	3.9 ± 2.5	3.1 ± 2.2	<0.001
25-OHD (ng/dL)	14.4 ± 8.1	18.6 ± 9.5	0.002
ALT (U/L)	25.8 ± 15.8	17.2 ± 13.2	0.002
Triglyceride (mg/dL)	112.7 ± 52.4	86.1 ± 40.7	0.002
LDL cholesterol (mg/dL)	103.2 ± 26.4	90.6 ± 22.9	0.010
HDL cholesterol (mg/dL)	49.4 ± 14	54.7 ± 14.5	0.034
Total cholesterol (mg/dL)	168.1 ± 30.7	160 ± 25.2	0.095

HOMA-IR: homeostatic model assessment of insulin resistance, 25-OHD: 25 hydroxyvitamin D.

**Table 3:** The correlation of HOMA-IR and vitamin D levels in obese group*.

25-OHD level (ng/dL)	<10 (n = 39)	10–20 (*n* = 43)	>20 (*n* = 31)	*P*
Male/female	19/20	22/21	16/15	>0.05
BMI	29.3 ± 3.4	29.3 ± 3.9	28.4 ± 4.2	>0.05
BMI SDS	2 ± 0.2	2 ± 0.2	2 ± 0.2	>0.05
HOMA-IR (mean ± SD)	3.9 ± 2.4	3.5 ± 2	3.8 ± 2.7	0.72

*One-way ANOVA test was used for correlation analysis.

**Table 4:** Bivariate correlations between covariates among HOMA-IR in the obese group.

HOMA-IR								
	Age (*n* = 118)	25-OHD (*n* = 112)	BMI (*n* = 115)	BMI SDS (*n* = 115)	BMI% (*n* = 115)	Triglycerides (*n* = 115)	LDL (*n* = 114)	HDL (*n* = 114)
**r	−0.041	−0.008	0.412	0.339	0.322	0.365	0.829	0.142
*P	0.664	0.935	<0.001	<0.001	<0.001	<0.001	<0.001	0.130

**Correlation is significant at the 0.01 level (2 tailed).

*Correlation is significant at the 0.05 level (2 tailed).

SDS: standard deviation score, BMI: body mass index, HOMA-IR: homeostatic model assessment of insulin resistance, 25-OHD: 25-hydroxyvitamin D level, LDL: low-density lipoprotein, and HDL: high-density lipoprotein.
